# MTEP, a Selective mGluR5 Antagonist, Had a Neuroprotective Effect but Did Not Prevent the Development of Spontaneous Recurrent Seizures and Behavioral Comorbidities in the Rat Lithium–Pilocarpine Model of Epilepsy

**DOI:** 10.3390/ijms23010497

**Published:** 2022-01-02

**Authors:** Alexandra V. Dyomina, Anna A. Kovalenko, Maria V. Zakharova, Tatiana Yu. Postnikova, Alexandra V. Griflyuk, Ilya V. Smolensky, Irina V. Antonova, Aleksey V. Zaitsev

**Affiliations:** Laboratory of Molecular Mechanisms of Neural Interactions, Sechenov Institute of Evolutionary Physiology and Biochemistry of RAS, 194223 Saint Petersburg, Russia; adyomina513@gmail.com (A.V.D.); kovalenko_0911@mail.ru (A.A.K.); zaharova-masha@yandex.ru (M.V.Z.); tapost2@mail.ru (T.Y.P.); griflyuk.al@mail.ru (A.V.G.); smolensky.ilya@gmail.com (I.V.S.); risha.irina999@mail.ru (I.V.A.)

**Keywords:** temporal lobe epilepsy, excitatory amino acid transporter 2, glial fibrillary acidic protein, open field test, novel object recognition test, hippocampus, immunohistochemistry, neuronal loss

## Abstract

Metabotropic glutamate receptors (mGluRs) are expressed predominantly on neurons and glial cells and are involved in the modulation of a wide range of signal transduction cascades. Therefore, different subtypes of mGluRs are considered a promising target for the treatment of various brain diseases. Previous studies have demonstrated the seizure-induced upregulation of mGluR5; however, its functional significance is still unclear. In the present study, we aimed to clarify the effect of treatment with the selective mGluR5 antagonist 3-[(2-methyl-1,3-thiazol-4-yl)ethynyl]-pyridine (MTEP) on epileptogenesis and behavioral impairments in rats using the lithium–pilocarpine model. We found that the administration of MTEP during the latent phase of the model did not improve survival, prevent the development of epilepsy, or attenuate its manifestations in rats. However, MTEP treatment completely prevented neuronal loss and partially attenuated astrogliosis in the hippocampus. An increase in excitatory amino acid transporter 2 expression, which has been detected in treated rats, may prevent excitotoxicity and be a potential mechanism of neuroprotection. We also found that MTEP administration did not prevent the behavioral comorbidities such as depressive-like behavior, motor hyperactivity, reduction of exploratory behavior, and cognitive impairments typical in the lithium–pilocarpine model. Thus, despite the distinct neuroprotective effect, the MTEP treatment was ineffective in preventing epilepsy.

## 1. Introduction

Temporal lobe epilepsy (TLE) is a severe neurological disorder, which manifests with spontaneously recurrent seizures and, in many cases, is accompanied by psychiatric comorbidities, such as depression, anxiety, psychosis, and cognitive impairment [1,2,3]. Often, it is difficult to find a treatment for this type of epilepsy, and almost 30% of cases are resistant to drug therapy [4,5]. However, since TLE is frequently the result of a primary brain injury, preventing TLE development following injury is considered an optimal therapeutic strategy for the acquired TLE [6,7].

However, a preventive treatment for epilepsy still does not exist [5]. Epileptogenesis refers to the sequence of events that converts the normal brain into one that can support a seizure. Seizures are thought to occur when the mechanisms that generally create a balance between excitation and inhibition are disturbed [8].

Metabotropic glutamate receptors (mGluRs) are not directly involved in fast excitatory synaptic transmission, unlike the ionotropic α-amino-3-hydroxy-5-methyl-4-isoxazole propionic acid (AMPA), *N*-Methyl-d-aspartate (NMDA), and kainite receptors. However, being present on the membranes of pre- and postsynaptic neurons and glial cells, they can modulate synaptic transmission, the membrane properties, and the metabolism of different cell types, thus, playing a crucial role in the balance of excitation and inhibition [9]. Moreover, mGluRs are also involved in the pathogenesis of several neurological and psychiatric disorders such as Alzheimer’s disease, Parkinson’s disease, anxiety, depression, and schizophrenia [10]. Group I and II mGluR-targeted drugs show beneficial effects in these conditions [11,12].

Eight mGluR subtypes are divided into three groups based on sequence homology, G-protein binding, and ligand selectivity [10]. Group I includes mGluR1 and 5, linked to G_q_/G_11_ proteins. The activation of these receptors leads to increased intracellular Ca^2+^ concentration and promotes NMDA receptor activation [13]. Group II (mGluR2, mGluR3) and III (mGluR4, mGluR6-8) receptors are coupled to G_i_/G_o_ proteins. The main effect of activating these receptors is a decreased neurotransmitter release and NMDA receptor activity [9,10].

Group I mGluR expression has previously been shown to be increased in the hippocampus in patients with epilepsy and a rat epilepsy model [14,15,16], indicating that this group of mGluRs may contribute to seizure susceptibility. In addition, agonists acting on mGluR1 or mGluR5 are convulsant; accordingly, negative modulators of group I mGluRs may be promising drugs for epilepsy treatment [17,18].

The selective mGluR1 antagonist, LY456236, exerts dose-related anticonvulsant effects for limbic seizures in the 6-Hz focal seizure model and the inhibition of tonic extensor seizures in the threshold electroshock model, suggesting that a mGluR1 blockade may be a clinically useful approach to treating epilepsy [19]. The mGluR5 antagonist, 2-Methyl-6-(phenylethynyl)pyridine (MPEP), produced dose-dependent protection against tonic extension seizures and the 6-Hz seizure test in mice [20] protected against sound-induced seizures in a mouse model of Fragile X syndrome [21]. However, no significant anticonvulsant activity was observed with the highly selective mGluR5 antagonist 3-[(2-methyl-1,3-thiazol-4-yl)ethynyl]-pyridine (MTEP) in the 6-Hz electroshock model [22].

Using the kainite model of epilepsy, Umpierre et al. (2019) showed that the mGluR5 expression in astrocytes could determine epilepsy development and modulate the interaction between astrocytes and neurons [23]. MTEP exhibited a robust neuroprotective effect against excitotoxicity induced by the intrahippocampal administration of kainic acid in rats [24]. MTEP was neuroprotective even if applied 1–6 h after the onset of exposure to kainic acid; however, the authors did not investigate its antiepileptogenic effect [24]. Moreover, MTEP administration has anxiolytic [25] and antidepressant effects [26], which might be applicable for these comorbidities in the epileptic models [27].

Therefore, we hypothesized that a mGluR5 blockade could significantly modify epileptogenesis, produce a neuroprotective effect, and reduce spontaneous recurrent seizures (SRSs) and comorbid behavioral impairments. The specific aim of the present study was to clarify the impact of MTEP treatment on epileptogenesis in the rat lithium–pilocarpine model. We chose this model since it is considered to be the most appropriate model of epileptogenesis and TLE [28,29]. Furthermore, it reproduces the main features of the pathological condition: (i) localization of seizure foci in the temporal lobe [30]; (ii) an “initial precipitating injury” which often precedes the onset of TLE [31]; (iii) a latent period without seizures [32].

## 2. Results

### 2.1. Overview of the Experimental Design

**Figure 1:**
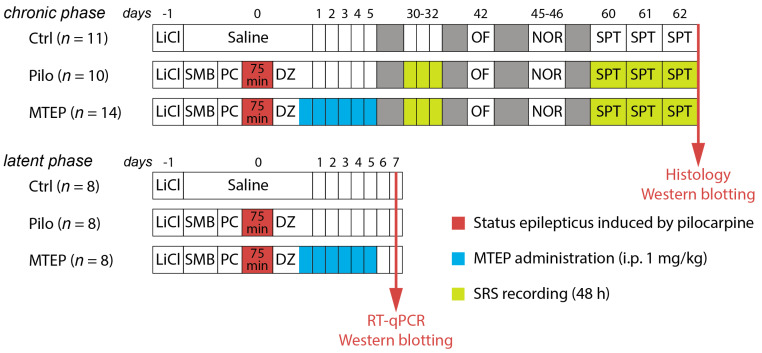
Experimental design. Pilo: rats administered with pilocarpine, MTEP: rats administered with pilocarpine and then treated with MTEP, LiCl: lithium chloride, SMB: (−)-scopolamine methyl bromide, DZ: diazepam, OF: open field test, NOR: novel object recognition, SPT: sucrose preference test, SRS: spontaneous recurrent seizure, qPCR: reverse transcription followed by a quantitative polymerase chain reaction, WB: Western blotting.

In this study, we administered pilocarpine in doses of 10 mg/kg every 30 min until the rat exhibited rearing (see Methods for details). Rats without rearing after administration of 40 mg/kg pilocarpine were excluded from the experiment. We stopped seizures by diazepam injection (5 mg/kg) 75 min after rearing started. MTEP (1 mg/kg) was administered 90 min after rearing and then every 24 h for 5 days in the experimental group. We performed RT-qPCR and Western blot analyses of some target genes/proteins in the rats’ hippocampus 7 days after status epilepticus (SE) during the latent phase of the model. Behavioral testing, SRS recording, histological analysis, and Western blot analyses were performed in the chronic phase of the model. The entire experimental design of this study is shown in Figure 1.

### 2.2. MTEP Administration Did Not Affect Rats’ Condition after Status Epilepticus and Spontaneous Recurrent Seizures

**Figure 2:**
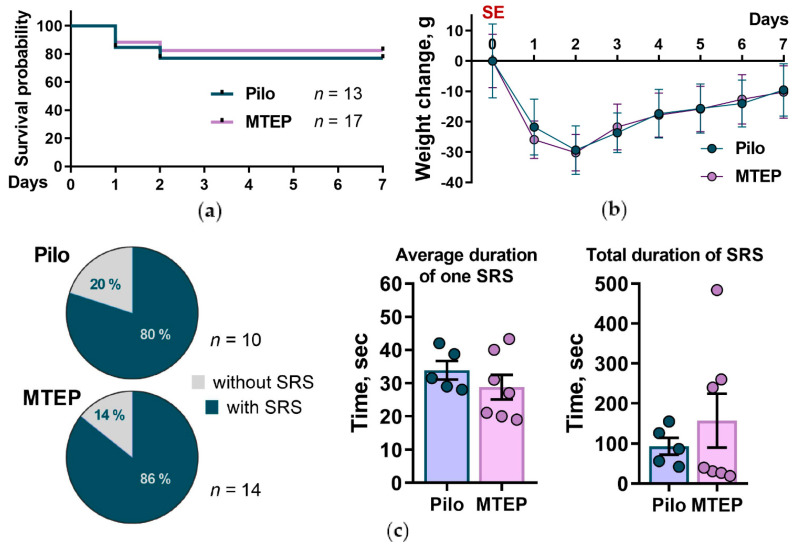
Survival and body weight dynamics in rats after lithium–pilocarpine induced status epilepticus (SE). (**a**) The Kaplan–Meier survival curves show no effect of MTEP treatment on survival (Gehan–Breslow–Wilcoxon test ꭓ^2^ = 1.3, *p* = 0.72). (**b**) Bodyweight dynamics are presented as alterations in weight relative to the average weight before SE. Only the rats that survived during the latent phase were used for bodyweight dynamics analysis (Pilo: *n* = 10; MTEP: *n* = 14). The data are presented as mean values with standard errors of the mean. Mixed ANOVA analysis showed no MTEP influence on weight dynamics (Greenhouse–Geisser F_2_._2,53_._9_ = 0.5, *p* = 0.7, η*_p_*^2^ = 0.02). (**c**) Spontaneous recurrent seizure (SRS) characteristics. The percentages of rats with SRS in the Pilo and MTEP groups do not differ; data includes video-recorded seizures and attacks noted during behavioral testing (left panel). The average duration of video recorded SRS (middle panel) and total SRS time (right panel) are not affected by MTEP treatment (student’s *t*-test, *p* > 0.05 for both parameters). The bars represent mean values, the error bars represent standard errors of the mean, and the circles represent the individual values of each animal.

To assess whether MTEP administration modifies the acute and latent phases of the model, we analyzed the survival rate and body weight of rats for one week after induced SE. We observed no differences between the Pilo and MTEP groups (Figure 2a,b).

To check if MTEP therapy had the anti-epileptogenic effect, we filmed the rats’ behavior for 48 h 1 and 2 months after SE. We analyzed the number and duration of SRS episodes (Figure 2c). Almost all animals in both groups exhibited SRSs, indicating that pilocarpine administration effectively induced epilepsy in rats. Some animals from both groups had seizures only during handling or behavioral tests but not during video recording. Therefore, we did not use these data to estimate the duration of SRSs. The percentage of rats with SRSs was the same in the Pilo and MTEP groups. We also noted that both groups exhibited equal SRS duration and frequency (Figure 2c). Thus, MTEP administration did not prevent epilepsy in the lithium–pilocarpine model or attenuate its manifestations.

### 2.3. Neuroprotective Effect of MTEP in Rat Hippocampus

**Figure 3:**
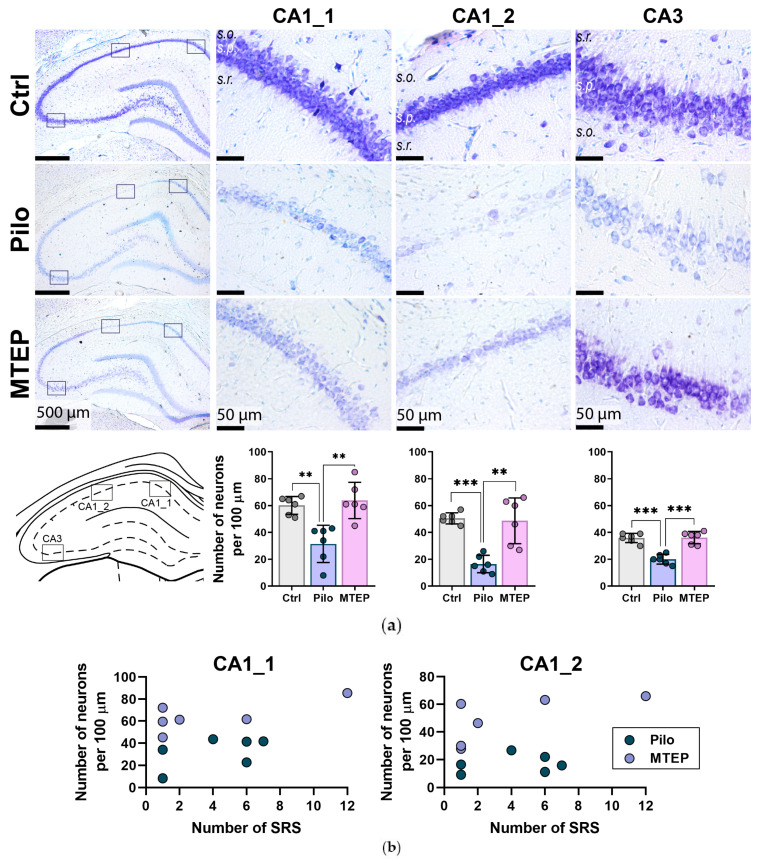
Nissl staining of rats’ hippocampus two months after SE showed the neuroprotective effect of MTEP therapy. (**a**) Three sites in the dorsal hippocampus were selected to analyze the number of neurons in the *str. pyramidale* (s.p.). CA1_1 is the most common site in the CA1 region for cell counting; CA1_2 is the place in the CA1 area with the most noticeable neurodegeneration. In the CA3 region, we counted neurons in the middle part of this area. The diagrams below show statistical data on the number of neurons per 100 µm length of the cellular layer. The circles show individual values per rat. The columns indicate average values and error bars show standard deviations. One-way ANOVA was performed to determine the neuroprotective effect of MTEP therapy. For CA1_1 region F_2,15_ = 13.4, *p* < 0.001, for CA1_2 F_2,15_ = 19.0, *p* < 0.001, for CA3 F_2,15_ = 30.6, *p* < 0.001. Asterisks indicate significant differences between groups according to Tukey’s post hoc tests: ** *p* < 0.01, *** *p* < 0.001. (**b**) Scatter plots illustrate the lack of correlation between the number of seizures and neuronal density in the CA1 area of the hippocampus. The circles show individual values.

Neurodegeneration in the dorsal hippocampus is often observed in the lithium–pilocarpine model [33,34]. MTEP demonstrated a significant neuroprotective effect in a kainate seizure model [24]. Therefore, we hypothesized that the neuroprotective effect of MTEP would also be observed in the lithium–pilocarpine model. We examined Nissl-stained hippocampal slices obtained two months after induced SE and detected a significant neuronal loss in the hippocampi of the Pilo rats, especially near the border of the CA1–CA2 areas, where we observed gaps in the cellular layer (Figure 3a).

The CA3 area looked more rarefied in the Pilo rats than in the controls. In the MTEP group, the neuronal loss in the hippocampus was less pronounced. We did not observe significant changes in neuron morphology in the epileptic animals compared with the controls. However, we noted a large number of small-nucleated cells in the CA1 pyramidal layer that were absent in the control hippocampi. Most likely, these small cells are astrocytes.

We counted the number of neurons in three different sites of the CA1 and CA3 areas (Figure 3a). The number of neurons in the Pilo group was 2–3 times lower than in the control and MTEP groups in the investigated regions (Figure 3a). Thus, MTEP administration during the latent phase prevented neuronal loss in the hippocampus in the lithium–pilocarpine model.

Next, we analyzed whether SRS frequency correlated with neuronal loss and used the CA1 sites because we observed the highest variability between rats at these places. However, we found no correlation between neuronal density and SRS frequency (Figure 3b). This result suggests that neuronal loss in the hippocampus is not the primary mechanism of epileptogenic focus formation.

### 2.4. MTEP Treatment Reduces Astrogliosis in the Epileptic Brain

Since astrocytes at epileptic tissue may contribute to seizure-inducing mechanisms [35,36] and the activation of astrocytic mGluR5s may increase basal synaptic transmission and neuronal synchrony [37], we investigated the effects of MTEP administration on astrocytes.

**Figure 4:**
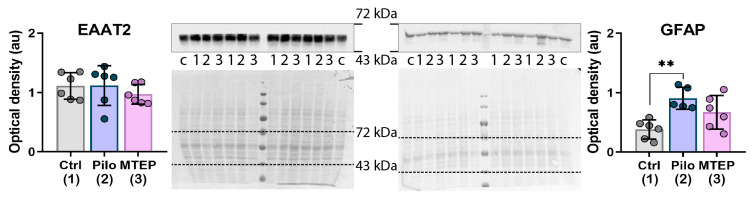
Western blotting data of proteins production in the dorsal hippocampus in the chronic phase. For inserts, the upper part shows the chemiluminescent signal, the lower part shows the Ponceau S. For bands, **c** is calibrator sample, **1** is Ctrl, **2** is Pilo, and **3** is MTEP group samples. Each dot represents one animal; the columns indicate average values and error bars show standard deviations. One-way ANOVA was used to determine the effect of MTEP therapy (excitatory amino acid transporter 2 (EAAT2): F_2, 17_ = 0,66, *p* = 0.53; glial fibrillary acidic protein (GFAP): F_2,16_ = 8.1, *p* < 0.01). Asterisks indicate significant differences between groups according to Tukey’s post hoc tests: ** *p* < 0.01.

First, we analyzed the expression levels of two astrocytic proteins, such as excitatory amino acid transporter 2 (EAAT2) and glial fibrillary acidic protein (GFAP), in the dorsal hippocampus using Western blotting analysis. One-way ANOVA revealed no significant changes in EAAT2 expression in hippocampi of epileptic animals (Figure 4), which is consistent with experimental and clinical data [38,39]. Additionally, MTEP treatment did not affect the expression level of EAAT2. In contrast, GFAP expression differed between groups (Figure 4). We found a significant increase in GFAP production in the Pilo group but not in the MTEP group (Figure 4).

**Figure 5:**
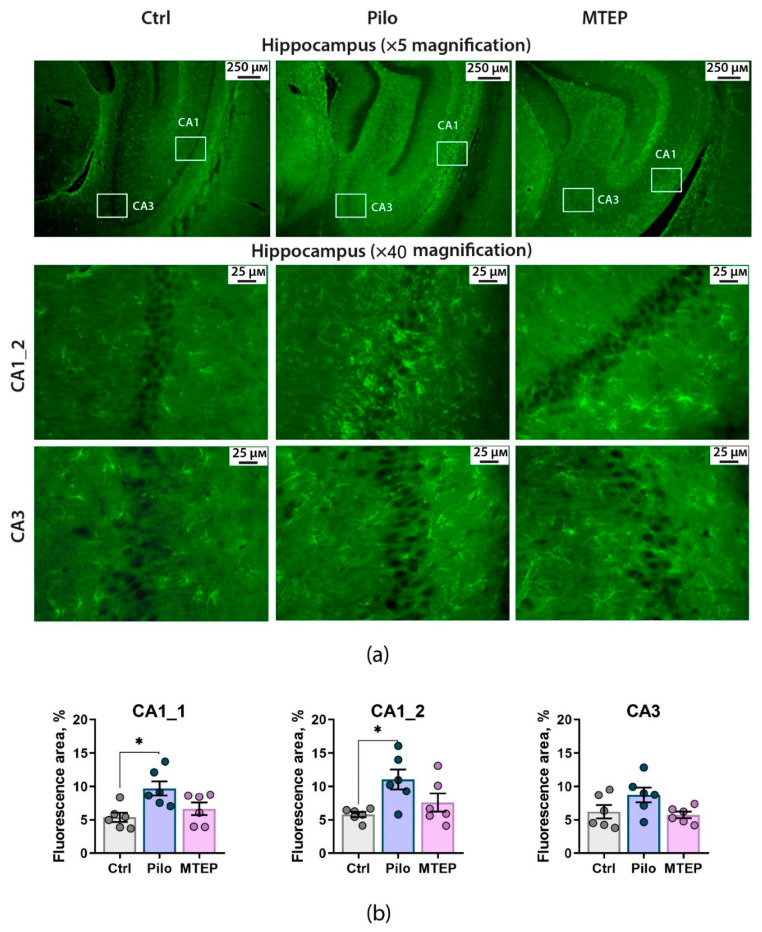
GFAP immunofluorescence analysis of the hippocampal tissue. (**a**) Representative images of hippocampal sections with GFAP-positive cells. Brain tissue was analyzed two months after pilocarpine-induced SE. Immunohistochemistry targeting glial fibrillary acidic protein (GFAP) was used to detect astrocytes. MTEP therapy reduces astrogliosis in the rat hippocampus. (**b**) The averaged GFAP-positive areas in different hippocampal sites: CA1_1, CA1_2, and CA3. The circles show individual values per brain. The columns indicate average values and error bars show standard errors of the means. One-way ANOVA was performed to determine the effects of MTEP therapy on astrogliosis: CA1 (F_2,15_ = 5.9; *p* < 0.05); CA2-CA1 (F_2,15_ = 8.4; *p* < 0.05); CA3 (F_2,15_ = 4.95; *p* = 0.07). Asterisks indicate significant differences between groups according to Tukey’s post hoc tests: * *p* < 0.05.

To clarify how GFAP production localized in the dorsal hippocampus, we performed GFAP immunofluorescence analysis of the hippocampal tissue (Figure 5). We found that in the epileptic brain, the distribution of astrocytes in the hippocampal layers was altered. The difference with the controls was most pronounced in the CA1 area, where the most remarkable neuronal loss was observed. Astrocytes migrate to the *stratum pyramidale*, and their processes overlap. In the CA3 area, the differences between the control and epileptic animals were less noticeable.

Next, we quantified the GFAP-positive areas in the control, Pilo, and MTEP groups (Figure 5b). One-way ANOVA revealed a significant effect of epilepsy on this parameter for both sites in the CA1 area (CA1_1: F_2,15_ = 5.9; *p* < 0.05; CA1_2: F_2,15_ = 8.4; *p* < 0.05) but not for the CA3 area (F_2,15_ = 4.95; *p* = 0.07). Tukey’s post hoc test revealed significant differences in the GFAP-positive area in CA1 between the control and Pilo groups only. No difference was found between the control and MTEP groups, although neither was found between the Pilo and MTEP groups. Thus, MTEP administration partially prevented astrogliosis in the epileptic brain.

### 2.5. MTEP Treatment Prevents a Decrease in EAAT2 Production but Does Not Affect an Increase in GFAP Expression in the Latent Phase of the Model

To understand why MTEP administration had a neuroprotective but not antiepileptogenic effect, we performed RT-qPCR and Western blot analyses of some target genes/proteins in the hippocampus of rats 7 days after SE. Since the leading cause of neuronal death after SE is excitotoxicity [40], we focused on glutamate reuptake and the expression level of ionotropic glutamate receptors.

**Figure 6:**
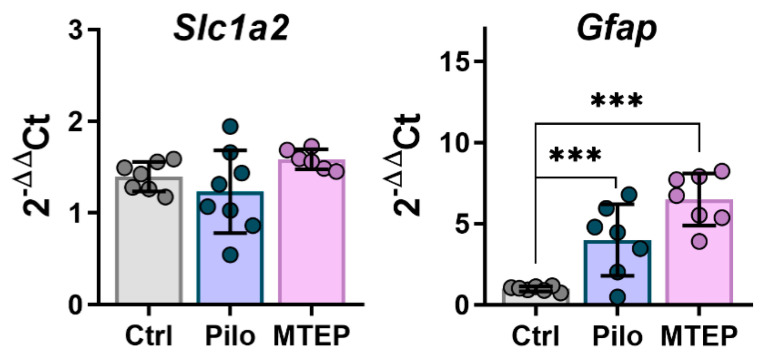
The relative expression of glial markers (*Slc1a2* and *Gfap*) in the dorsal hippocampus 7 days after pilocarpine-induced SE. Each dot represents one animal; the bars indicate average values and error bars show standard deviations. One-way ANOVA: *Slc1a2:* F_2,21_ = 1.4, *p* = 0.3; *Gfap:* F_2,20_ = 21.5, *p* < 0.001. Asterisks indicate significant differences between groups according to Tukey’s post hoc tests: *** *p* < 0.001.

**Figure 7:**
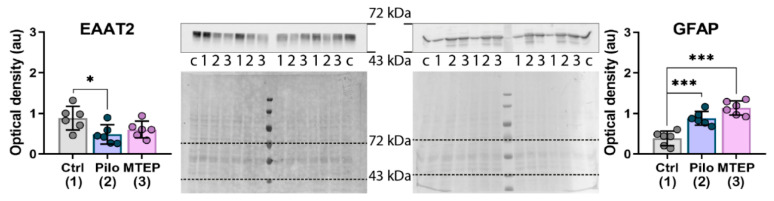
Western blotting data of proteins production in the dorsal hippocampus 7 days after pilocarpine-induced SE. For inserts, the upper part shows the chemiluminescent signal, the lower part shows the Ponceau S. For bands **c** is sample calibrator, **1** is Ctrl, **2** is Pilo, and **3** is MTEP group. Each dot represents one animal; the columns indicate average values and error bars show standard deviations. One-way ANOVA, GFAP: F_2,17_ = 29, *p* < 0.001; EAAT2: F_2,17_ = 4, *p* < 0.05. Asterisks indicate significant differences between groups according to Tukey’s post hoc tests: * *p* < 0.05, *** *p* < 0.001.

While there is a molecular diversity of glutamate transporters, in the hippocampus, ~80–90% of glutamate reuptake is provided by EAAT2 [41]. Most previous studies revealed a decrease in EAAT2 expression during the latent phase of different drug-induced models of epilepsy [42,43], which can lead to glutamate excess and excitotoxicity. In contrast, EAAT2 over-expression is one of the effective neuroprotective mechanisms [41,44]. Therefore, we analyzed the expression of EAAT2 and a glial marker, GFAP, on mRNA (*Slc1a2* and *Gfap* genes, respectively, Figure 6) and protein levels (Figure 7). We found a significant decrease in EAAT2 expression at the protein level but not at the mRNA level in the Pilo rats. MTEP therapy prevented a decline in EAAT2 protein expression.

Based on these results, we can assume that MTEP administration might provoke short-term glial activation and prevent a decline in EAAT2 protein production. Thus, maintenance of EAAT2 expression with MTEP may be one of the mechanisms of neuroprotection.

### 2.6. Changes in Gene Expression and Protein Production of Ionotropic Glutamate Receptors

**Figure 8:**
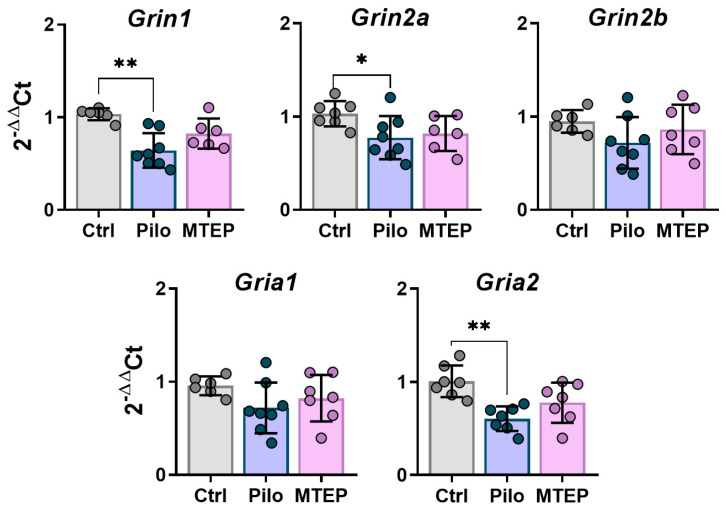
The relative expression of genes of NMDA and AMPA receptor subunits in the dorsal hippocampus 7 days after pilocarpine-induced SE. Subunit genes: *Grin1*–GluN1, *Grin2a*–GluN2a, *Grin2b*–GluN2b, *Gria1*–GluA1, *Gria2*–GluA2. Each dot represents one animal; the bars indicate average values and error bars show standard deviations. One-way ANOVA, *Grin1:* F_2,19_ = 11.8, *p* < 0.001; *Grin2a:* F_2,20_ = 3.7, *p* < 0.05; F_2,20_ = 1.7, *p* = 0.2; *Gria1:* F_2,20_ = 1.8, *p* = 0.2; *Gria2:* F_2,20_ = 9.1, *p* < 0.01. Asterisks indicate significant differences between groups according to Tukey’s post hoc tests: * *p* < 0.05; ** *p* < 0.01.

Next, we analyzed changes in the gene expression of NMDA and AMPA receptors in the hippocampus (Figure 8). We found that the expression levels of *Grin1, Grin2a,* and *Gria2* (GluN1, GluN2a, and GluA2 subunits) were reduced in the Pilo rats. However, MTEP administration restored the expression level of ionotropic receptor subunits. Thus, MTEP treatment minimized changes in glutamate receptor gene expressions after pilocarpine-induced SE.

**Figure 9:**
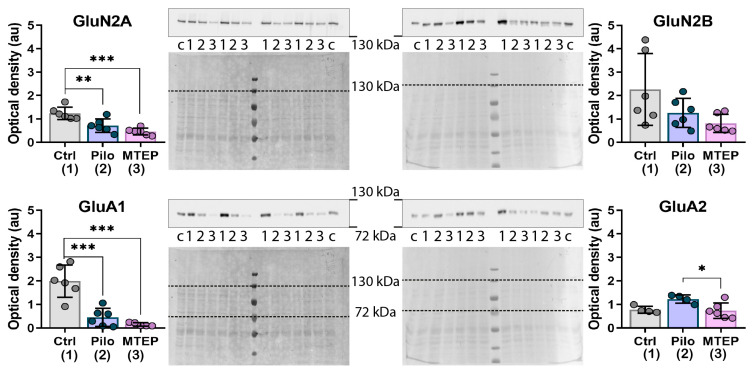
Western blotting data of proteins production in the dorsal hippocampus 7 days after pilocarpine-induced SE. For inserts, upper part in each line shows the chemiluminescent signal, lower part shows the Ponceau S. For bands **c** is calibrator sample, **1** is Ctrl, **2** is Pilo, and **3** is MTEP group. On charts each dot represents one animal; the columns indicate average values, and error bars show standard deviations. One-way ANOVA, GluN2A: F_2,17_ = 16, *p* < 0.001; GluN2B: F_2,8_._62_ = 3.04, *p* = 0.10; GluA1: F_2,16_ = 25.4, *p* < 0.001, GluA2: F_2,13_ = 5.1, *p* < 0.05. Asterisks indicate significant differences between groups according to Tukey’s post hoc tests: * *p* < 0.05; ** *p* < 0.01, *** *p* < 0.001.

**Figure A1:**
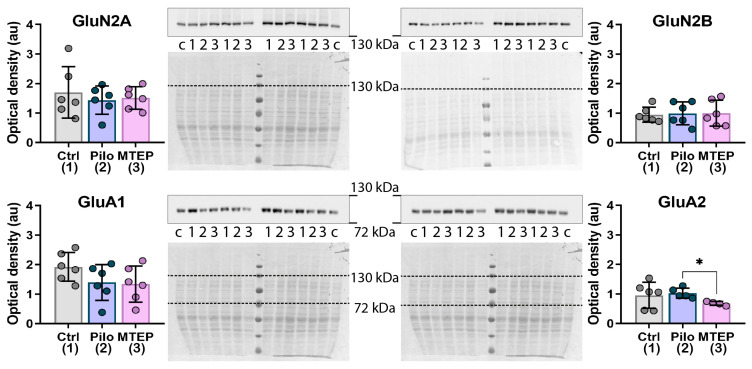
Western blotting data of proteins production in the dorsal hippocampus in chronic phase. For inserts, the upper part shows the chemiluminescent signal and the lower part shows the Ponceau S. For bands, **c** is calibrator sample, **1** is Ctrl, **2** is Pilo, and **3** is MTEP group. On charts, each dot represents one animal; the columns indicate average values, and error bars show standard deviations. One-way ANOVA, GluN2A: F_2,17_ = 0.3, *p* = 0.75; GluN2B: F_2,17_ = 0.3, *p* = 0.99; GluA1: F_2,16_ = 1.9, *p* = 0.19, GluA2: F_2,7_._1_ = 8.7, *p* = 0.012. Asterisks indicate significant differences between groups according to Games–Howell post hoc tests: * *p* < 0.05.

We performed a Western blot analysis to check whether gene expression characteristics are reproduced by protein production. Consistent with the gene expression results, we found decreased production of GluN2a subunits in the Pilo rats (Figure 9). However, MTEP treatment had no beneficial effect on GluN2a production. We found a significant decrease in GluA1 subunit production in the Pilo and MTEP rats that was not detected by gene expression. Interestingly, GluA2 production was lower in the MTEP group than in the Pilo group, and the declined production of GluA2 in the MTEP group persisted in the chronic phase (Figure A1).

### 2.7. Behavioral Alterations in Chronic Phase of the Model

The lithium–pilocarpine model is accompanied by the profound alteration of animal cognitive and emotional behavior [27,45]. We suggested that MTEP treatment might prevent some behavioral comorbidities because mGluR5s participate in mood disorder regulation [46]. Therefore, we ran a series of behavioral tests in the chronic phase of the model.

#### 2.7.1. MTEP Treatment Does Not Prevent Depressive-Like Behavior in Epileptic Rats

**Figure 10:**
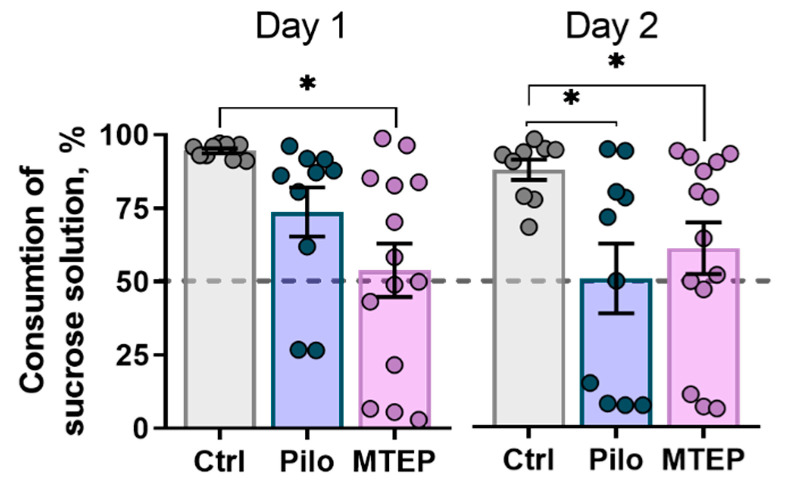
Sucrose solution consumption in a sucrose preference test. Welch ANOVA was used for statistical analysis–Day 1: F_2,14_._4_ = 12.3, *p* < 0.001; Day 2: F_2,16_._6_ = 7.4, *p* < 0.01. The circles show individual values. The columns indicate average values and error bars show standard errors of the means. Asterisks indicate differences between groups according to Games–Howell post hoc test: * *p* < 0.05.

First, we tested if the MTEP therapy had a long-term antidepressant-like effect. To detect signs of depressive-like behavior in rats two months after SE, we measured the level of anhedonia in the sucrose preference test. We found reduced sucrose solution consumption in both the Pilo and MTEP groups compared with the control group (Figure 10). Interestingly, in the MTEP group, significant anhedonia had already been observed on the first day of the test, suggesting that depressive-like behavior was even more prominent in this group.

#### 2.7.2. MTEP Treatment Changed Preference for Areas in Open Field Test but Did Not Influence Motor Hyperactivity

**Figure 11:**
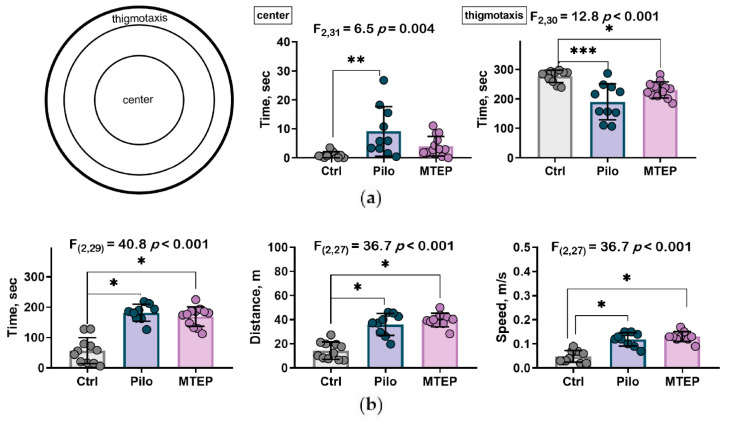
Open field test. (**a**) Distribution of time spent in center and thigmotaxis areas of the open field. (**b**) Locomotion characteristics in the open field test. * *p* < 0.05, ** *p* < 0.01, *** *p* < 0.01 in one-way ANOVA with Tukey’s post hoc test.

Preference for a specific area in the open field can characterize anxiety in rodents [47,48]. We analyzed time spent in the center and the thigmotaxis area to reveal anxiety-like behavior. The control rats spent almost all their time in the thigmotaxis area and the Pilo rats spent only half their time there (Figure 11a). In the MTEP group, this parameter approached the level of the control group but was still lower. The Pilo rats crossed the open field center more frequently than the rats from the control and MTEP groups.

These results mean that anxiety patterns in the open field were disturbed in the Pilo rats, and MTEP administration partially restored the rats’ behavior.

To check the impact of MTEP treatment on locomotor hyperactivity that was previously found in the model [27], we analyzed motor activity parameters. We found that the Pilo rats spent more time in motion and, at the same time, moved faster than the control rats (Figure 11b). Furthermore, the MTEP rats exhibited the same activity parameters as the Pilo rats, suggesting that MTEP does not modify hyperactive behavior.

#### 2.7.3. Reduction of Exploratory Behavior and Cognitive Impairments

**Figure 12:**
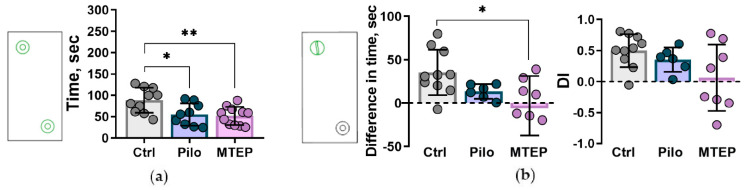
Exploratory behavior and memory characteristics in the novel object recognition test. (**a**) The novel objects interaction paradigm: chart shows time spent interacting with identical objects. * *p* < 0.05, ** *p* < 0.01 in one-way ANOVA with Tukey’s post hoc test, F_2,27_ = 6.4, *p* = 0.005. (**b**) Novel object recognition: the left chart shows the difference in time spent on interaction with novel object versus familiar object, the right chart shows discrimination index (DI) for which +1 indicates a preference of the novel object, −1 indicates a preference of the familiar object, and 0 indicates no preferences. * *p* < 0.05 in one-way ANOVA with Tukey’s post hoc test, F_2,21_ = 4.8, *p* = 0.02. On all charts, the circles show individual values. The columns indicate average values and error bars show standard deviations.

We ran a novel object interaction paradigm to estimate exploratory behavior before cognitive testing [49]. First, we put the rat into a familiar Plexiglas box with two identical novel objects. We found that the Pilo and MTEP rats spent less time exploring new objects than the control animals (Figure 12a). This fact indicates a decrease in exploratory behavior in epileptic rats.

Next, we applied a novel object recognition task in a familiar environment to test short-term memory. On the first attempt with the identical objects, we counted the total time spent on interaction with one of the identical objects (object A), in the place of which there would be a novel object later. There were no significant differences between the groups. In the second attempt, object A was replaced with a novel object, and we calculated the total interaction time with the novel object. In the control group, time spent interacting with the novel object was more prolonged than in the other groups (one-way Welch’s ANOVA, F_2, 14_._9_ = 5.7, *p* < 0.001 with Games–Howell post hoc test *p* < 0.05). The control rats investigated the novel object longer than the interaction with object A (repeated measures ANOVA F_4, 44_ = 3.6, *p* < 0.05, between successive attempts in Control group T_9_ = 2.53, *p* = 0.032 in paired sample *t*-test), that was not observed in the other two groups.

Analysis of the novel object preference by the difference in time spent on the novel object versus the familiar object showed a significant lack of interaction with the novel object in the MTEP group (Figure 12b). The discrimination index also reveals no preference in the MTEP group. These results indicate a disturbance in the ability to recognize novel objects or working memory functions.

## 3. Discussion

In this study, we investigated the effects of MTEP administration on epileptogenesis during the latent period of the lithium–pilocarpine model of epilepsy. We measured neurological conditions during the latent and chronic phase and found that MTEP did not influence body weight dynamics, survival, and SRS formation, which indicates the absence of antiepileptic effects of MTEP treatment. Nevertheless, we found a prolonged neuroprotective effect of MTEP treatment in the rat hippocampus afterward. A previous study demonstrated only an acute neuroprotective effect of MTEP administration in the kainite seizure model [24].

Another finding of this study is that MTEP administration partially prevents astrogliosis in the rat hippocampus. Hippocampal sclerosis is characteristic damage of the model [50,51].

In this study, we tested the effect of a small dose of MTEP (1 mg/kg), which has been previously shown to have a neuroprotective effect in the kainate-induced excitotoxicity model [24]. The anticonvulsant effect of high doses of MTEP (20–40 mg/kg) has been shown in immature rats [52]. However, high doses of MTEP have pronounced side effects [53,54]. To our knowledge, no studies have investigated the antiepileptogenic effect of MTEP at any dose, so we used MTEP only in a low amount in our study.

We tried to determine the mechanism of the neuroprotective action of MTEP. It is known that mGluRs are expressed primarily on neurons and glial cells and participate in the modulation of a wide range of signal transduction cascades [55,56]. We hypothesized that the use of MTEP could affect the expression of astroglial proteins and different subunits of NMDA- and AMPA-receptors at the latent and chronic phases of the model. The production of GFAP was increased in the hippocampus of the Pilo rats in line with previous studies in the lithium–pilocarpine model [34,57]. However, the MTEP treatment had almost no effect on this change.

In contrast, MTEP treatment prevented reducing the expression of another astroglial protein, EAAT2. Reduced EAAT2 production may contribute to excitotoxicity and neuronal death, as glutamate remaining in the synaptic cleft will lead to the over-activation of its receptors [40]. In this regard, the change in EAAT2 protein levels that we have identified may be one of the mechanisms of epileptogenesis [58] and is consistent with previous findings [59]. Furthermore, in our study, MTEP administration contributed to the maintenance of EAAT2 expression at control levels during the latent phase of the model, which may be one of the neuroprotection mechanisms.

Epilepsy is known to be characterized by a rearrangement of the subunit composition of ionotropic glutamate NMDA- and AMPA-receptors [60,61,62,63,64], which may be a factor in epileptogenesis and the development of neurological abnormalities [27,65,66,67]. We found decreased gene expression of NMDA (Grin1, Grin2a) and AMPA (Gria2) receptor subunits in the Pilo group, which is consistent with previously published results [61,68,69]. MTEP administration mainly maintained the gene expression of ionotropic receptor subunits at control levels in the hippocampus. However, MTEP reduced GluA2 expression at the protein level in both latent and chronic phases, while no reduction in this subunit was observed in the Pilo group. A decrease in GluN2a and GluA1 production was detected in the Pilo and MTEP groups only in the latent phase. Thus, MTEP treatment minimized ionotropic glutamate receptor gene expression disruption, but there was no significant effect at the protein level.

MTEP administration within the latent phase reduced astrogliosis in the hippocampus in the chronic phase. This could potentially indicate a weakening of the epileptic processes. For example, astrocytes activation in human temporal lobe epilepsy is associated with refractory disease forms [70].

mGluR5 takes on special significance during epileptogenesis and promotes the reproduction of astrocyte–neuron interaction patterns characteristic of the developing brain, allowing more effective synaptic glutamate clearance [23]. Furthermore, mGluR5 regulates glial physiological sensitivity to glutamate and activity in pathological conditions [37,71]. However, the effects of activating or inhibiting mGluR5 on glial cells and neurodegeneration can be quite complex and depend on the model used. Positive modulation of mGluR5 at a delayed stage of the traumatic brain injury model can prevent neuroinflammation and neurodegeneration [72] In that model, the administration of (RS)-2-chloro-5-hydroxyphenylglycine (CHPG), an agonist of mGluR5, reduced expression of reactive microglia expressing NADPH oxidase subunits and decreased hippocampal neuronal loss. Our study did not reveal a decrease in astrocytes activation by MTEP treatment. However, we observed the reduced astrogliosis as a delayed effect, which has not been previously shown.

Metabotropic glutamate receptors take part in mood regulation and disorders [46], so MTEP therapy in the latent phase might influence the behavior comorbidities in the model. We expected a correction of depressive-like behavior in the model by MTEP because of its antidepressant-like effects [73,74], but that did not happen. It might be due to the involvement of NMDA receptors in implementing the acute MTEP effect [75].

A depressive-like behavior manifests in the chronic phase of the model [76], in which MTEP did not influence NMDA activity. On the other hand, the antidepressant-like effect of MTEP was shown in the forced swimming test, and some authors claim that the immobility time that this test reflects is not the depressive-like behavior but the passive stress coping strategy [77]. In line with this assumption, our results of the open field test show that healthy rats prefer to remain close to vertical surfaces (walls in the open field, [78]), while the Pilo rats walk across the whole arena. The typical behavioral patterns of the MTEP-treated rats were restored. Usually, an increase in time spent in the center is interpreted as a decrease in anxiety level [47,48]. However, our previous results [27] suggest that these results should be interpreted in terms of changes in the stress-coping strategy. Although the Pilo-rats exhibited a less anxious phenotype, they still possessed high-stress reactivity. Considering all outcomes, we are inclined to talk about changes in behavioral strategies, but we cannot unequivocally assess the susceptibility to stress and differences in anxiety levels in rats.

In rats with temporal lobe epilepsy, it is supposed that low fidelity of spatial signals in the hippocampus network underlies cognitive dysfunctions [79]. Therefore, we expected cognitive functions to improve due to the neuroprotective effects of MTEP, but the novel object recognition test instead showed a deterioration in the already impaired memory. This behavior might be a consequence of the depressive-like phenotype of epileptic rats [19], which may be accompanied by cognitive impairment by itself [80]. Additionally, the lithium–pilocarpine model is characterized by the widespread damage of brain structures [45], so neuroprotection in the hippocampus may not be enough for improving cognitive function because it does not solve the problem of reduced network efficiency [81,82].

This study demonstrates that MTEP has a significant long-term neuroprotective effect and partially prevents astrogliosis. However, it is not efficient when used alone as a preventive treatment of epileptogenesis. Moreover, the MTEP therapy contributed to a more explicit manifestation of anxiety-depressive disorders in the model.

## 4. Materials and Methods

### 4.1. Animals

Male Wistar rats were housed in standard home cages (5–6 rats per cage) with free access to the food and water and 12 h dark–light cycle (dark at 8 p.m.–light at 8 a.m.). Rats in the control and experimental groups were mixed from different litters to avoid any influence from genetic factors. The experiments were conducted in compliance with the Rules of Animal Care and Use Committee of the Sechenov Institute of Evolutionary Physiology and Biochemistry of the RAS. These rules are fully compliant with the EU Directive 2010/63/EU for animal experiments.

### 4.2. Lithium–Pilocarpine Model and Treatment

Seven-week-old rats received lithium chloride (intraperitoneally (i.p.), 127 mg/kg; Sigma-Aldrich, St. Louis, MO, USA) one day before pilocarpine. One hour before pilocarpine injection, (−)-scopolamine methyl bromide (1 mg/kg, i.p., Sigma-Aldrich) was administrated to block the peripheral muscarinic receptors. Then rats received one or several injections of pilocarpine (i.p., Sigma-Aldrich), depending on the intensity of the induced seizures. Seizure severity was estimated according to modified Racine’s scale [27]: facial automatism (1), head-nodding (2), forelimb myoclonus (3), rearing (4), rearing and falling (5), wild running (6), generalized clonic-tonic convulsions (7). The first dose of pilocarpine was 10 mg/kg. If the rat did not exhibit convulsions of score 4 or above within 30 min, the additional doses of pilocarpine (10 mg/kg) were injected every 30 min. Rats that did not produce convulsions of score 4 or above after the fourth injection were excluded from further experiments. One hour and 15 min after the seizure of score 4 started, diazepam (3–5 mg/kg, i.p., Sigma-Aldrich) was administered to cease convulsions. The control rats received lithium chloride and saline only.

One and a half hours after diazepam administration, half of the rats were given MTEP (1 mg/kg, i.p., Sigma-Aldrich, MTEP group), all other rats received saline. Then injections were given once a day for five consecutive days (Figure 1). Such a dose of MTEP has been shown to have a neuroprotective effect [24].

### 4.3. Evaluation of Rats Condition after Status Epilepticus

Survival and body weight were monitored for 7 days after SE. At this time, rats were injected daily with 5% glucose solution (2 mL, subcutaneously) to increase survival.

### 4.4. Spontaneous Recurrent Seizures (SRSs)

The presence of SRS was assessed twice: 1 and 2 months after SE. Each rat was placed in an individual transparent cage for SRS detection with continuous video recording. We calculated the duration of each SRS episode and total seizure duration during video recording. Additionally, SRSs were noted in the database if they were observed during handling or behavioral testing.

A single SRS episode was determined as a motor seizure. Typically, SRS starts with facial automatism (rats usually freeze during this stage, it was considered as the 1st stage in Racine’s scale). Then, going sequentially through all (or only a few) steps, the rat could pass to rearing and falling (5th stage in Racine’s scale), sometimes with wild running (6th stage), and ended with the generalized clonic-tonic convulsions (7th stage) and then subsided. In any case, the end of the seizure was considered the return of the rat to regular motor activity. The duration of each SRS was measured only for videotaped episodes.

### 4.5. Behavioral Testing

Behavioral testing was performed in the chronic phases of the model (Figure 1). The results were excluded from the analysis if the rat exhibited spontaneous seizure during testing. We also made sure that there was no seizure half an hour before testing.

#### 4.5.1. Sucrose Preference Test

We used the sucrose preference test to estimate depressive-like behavior (anhedonia) [76,83]. The test was performed for two consecutive days. Rats were placed in individual cages (30 × 30 cm, height = 40 cm) with two bottles, the first of which contained normal drinking water and the second one 1% sucrose solution. Rats had free access to the bottles. Solution consumption was measured every day. The sucrose preference was calculated as the ratio of sucrose solution consumption to total liquid consumption.

#### 4.5.2. Open Field Test

The open field test was used to assess motor activity and anxiety [59]. The open field arena had a diameter of 1 m, wall height of 30 cm, and illumination in the center was 8 Lx. The rat was placed in the center of the arena, and then the rat’s movement was recorded for 5 min. The recordings were analyzed offline using Round and Cross software (Institute of Experimental Medicine, St. Petersburg, Russia). We calculated time spent in the different field zones (in the center of the arena (1/4 of arena radius) and near the wall (less than 20 cm from the wall, thigmotaxis) to estimate anxiety level. Total distance, time of locomotion, and velocity were calculated to determine locomotor activity.

#### 4.5.3. Novel Object Recognition Test and Novel Object Interaction Paradigm

**Figure 13:**
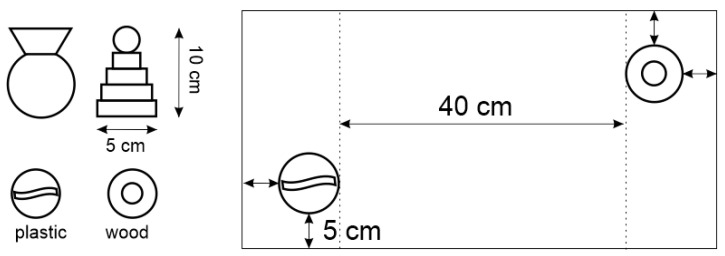
The objects for the novel object recognition test and their mutual arrangement in Plexiglas box.

The novel object interaction paradigm [49] and novel object recognition test [84] were used for the estimation of exploratory behavior and short-time memory, respectively. The testing was carried out in a Plexiglas box (60 × 30 cm and 40 cm high), which was under the upper camera. We used two pairs of toys as new objects (Figure 13). The toys were chosen so that the rat initially had no preference and could not climb or sit on them.

The day before testing, the rats were placed together in a Plexiglas box without toys for 24 h to reduce stress levels and exploratory activity in the box. The day before the experiment, the rats were placed back in their home cage.

The test consisted of two 5-min steps. In the first step, one of the pairs of identical toys was placed in a Plexiglas box. The rat was placed in the center of the cage, and its behavior was recorded for 5 min. The first step was also used as a paradigm of interaction with the new object. In the second step, one toy was replaced with a new one. All toys and the Plexiglas box were cleaned with 0.3% peroxide solution.

The recordings were analyzed offline using Field4W software (Institute of Experimental Medicine, St. Petersburg, Russia). We measured the time of interaction of each object: sniffing, groping, attempts to eat the toy. If the total interaction time was less than 15 s, the rat was excluded from the analysis. For the novel object interaction paradigm, the total time of interaction with both toys in the first step was analyzed. For the novel object recognition test, we measured the time of interaction with each toy. We then calculated the difference in interaction time for the novel versus familiar object and the discrimination index as a ratio of the difference to sum up the interaction time for the novel versus familiar toy [84].

### 4.6. Tissue Preparation and Nissl Staining

Rats were anesthetized with an Isoflurane (Laboratorios Karizoo, Barcelona, Spain) and decapitated 70 days after SE. Then, the brains were quickly removed, and one hemisphere was fixed in 4% PFA at 4 °C for 7 days. The other hemisphere was used for Western blot analysis. Next, brains were cryoprotected with 30% sucrose, frozen in cooled isopentane (−50 °C; 78–78-4, Isopentane Solution, Sigma-Aldrich), and stored at −80 °C.

The 20-μm-thick frontal serial sections (from −2.6 to −3.6 mm from the bregma) were cut on a cryostat Bright OTF5000 (Bright Instrument Co Ltd., Huntingdon, UK), Nissl-stained using thionin blue (Thionin powder, T-409, Fisher Chemicals) [85].

For morphological analysis, neuronal counts were performed on every fifth section (yielding 8–10 sections from one rat hippocampus). Sections were analyzed using the Leica Microscope AF 7000 (Leica Microsystems, Wetzlar, Germany) under ×400 magnification. The number of neurons in digital micrographs was counted per 100 μm for the cell layer in CA1 and CA3 using ImageJ (U.S. National Institutes of Health, Bethesda, MD, USA).

### 4.7. Immunohistochemistry

Sections were incubated in phosphate-buffered saline with 0.2% Triton X-100 (PBST) for 30 min, and then in blocking serum (2% normal goat serum and 3% bovine serum albumin in PBST) for 1 h. Next, sections were incubated with primary antibodies mouse anti-GFAP (1:1000 in PBST; NBP1-05197; Bio-Techne Ltd., Abingdon, United Kingdom) for 48 h at 4 °C and then with chicken anti-mouse IgG cross-adsorbed secondary antibody, Alexa Fluor 488 (1:500, A-21200; Thermo Fisher Scientific Inc., Waltham, MA, USA) for 2 h at room temperature.

Sections were analyzed using the Leica Microscope AF 7000 under ×400 magnification. The area (in %) occupied by GFAP-positive objects was assessed using ImageJ. Between 7 and 10 sections from one hippocampus were used for analysis.

### 4.8. mRNA Expression Analysis

Rats were decapitated 7 days after SE. The brain was quickly removed and frozen at −80 °C. The dorsal area of the hippocampus was dissected using the OTF5000 Cryostat Microtome (Bright Instruments, Luton, UK) according to the rat brain atlas [65]. Total RNA was extracted using the ExtractRNA reagent (Evrogen, Moscow, Russia) according to the manufacturer’s instructions. RNA samples were treated with 1 unit of RQ1 DNAse (Promega, Madison, WI, USA) for 15 min followed by 8 M LiCl (3 volumes of LiCl to 1 volume of RNA solution) precipitation and 75% ethanol washing. RNA concentration and purity were assessed spectrophotometrically based on a 260 nm absorbance and a 260/280 absorbance ratio, respectively, using the NanoDrop™ Lite Spectrophotometer (Thermo Fisher Scientific).

cDNA was synthesized from 1 μg of total RNA, with oligo-dT (0.5 µg per 1 µg of RNA) and 9-mer random (0.25 µg per 1 µg of RNA) primers (DNA Synthesis Ltd., Moscow, Russia) and M-MLV reverse transcriptase (100 units per 1 µg of RNA; Evrogen, Moscow, Russia) in a total volume of 20 µL following the manufacturer’s instruction. All samples were diluted 10-fold before the PCR step.

qPCR was performed in a total volume of 6 µL with 0.8 µL of cDNA, 0.5 units of TaqM-polymerase (Alkor Bio, St. Petersburg, Russia), 3.5 mM of Mg^2+^, and specific forward primers, reverse primers, and hydrolysis (TaqMan) probes (see Appendix A Table A1). Nucleotides were purchased from DNA Synthesis Ltd. (Moscow, Russia). qPCR reactions were multiplexed as follows: glial fibrillary acidic protein (*Gfap*) with solute carrier family 1 member 2 gene (*Slc1a2*, encoding excitatory amino acid transporter 2); subunits of NMDARs and AMPARs-*Grin1* + *Grin2a*, *Grin2b* + *Gria1* + *Gria2*; three triplex qPCR assays for reference genes *Actb* + *Gapdh* + *B2m*, *Rpl13a* + *Ppia* + *Sdha*, and *Hprt1* + *Pgk1* + *Ywhaz* as previously described [86]. PCR reactions were tetraplicated and carried out in a C1000 Touch thermal cycler combined with a CFX384 Touch™ Real-Time PCR Detection System (BioRad, Hercules, CA, USA) simultaneously with no template and no reverse transcription control samples. Reference genes for the normalization of expression data were selected based on comprehensive ranking obtained using the RefFinder online tool (https://www.heartcure.com.au/reffinder/ (accessed on 29 December 2021)) incorporated with the GeNorm [87], NormFinder [88], BestKeeper [89], and comparative deltaCT [90] algorithms. The relative expressions of the genes were calculated using the 2−ΔΔCt method [91] normalized against the geometric average for the three most stable reference genes (*Ppia*, *Ywhaz*, *Pgk1*).

### 4.9. Western Blotting

Rats were decapitated 7 or 60 days after seizure induction. After decapitation, brains were quickly isolated, frozen, and stored at −80 °C before dissection. Similar to the qPCR step, the dorsal hippocampus was dissected. Samples were homogenized in 150 µL of lysis buffer [92] containing 100 mM Tris–HCl pH 8.0, 140 mM NaCl, 20 mM EDTA, 5% SDS, 1× protease inhibitor cocktail (Pierce Protease Inhibitor Tablets, Thermo Fisher Scientific) and 1× phosphatase inhibitor cocktail (Phosphatase Inhibitor Cocktail II, Abcam, Cambridge, UK), then incubated for 1 h at room temperature with constant agitation. Following centrifugation (14,000× *g* for 10 min), the supernatant was used for protein quantification and further assay. Protein concentration was determined as described previously [93]. Protein supernatant was mixed 1:1 with 2× loading buffer (125 mM Tris–HCl pH 6.8, 40% glycerol, 4% SDS, 10% β-mercaptoethanol, 0.02% bromophenol blue) and heated at 7 °C for 15 min. Aliquots of 6 µg of protein in equal volumes were loaded onto a 7% polyacrylamide gel and separated by electrophoresis (125 V) under reducing and denaturing conditions [94] with a Thermo Scientific PageRuler Prestained Protein Ladder (10–170 kDa; Thermo Fisher Scientific) until the 34 kDa band of the ladder reached the gel border. Each gel contained a calibrator sample, which was prepared by mixing samples from each control and experimental group. The amount of loaded protein was determined as previously described [93]. After electrophoresis, proteins were transferred from the gel onto a 20 µm nitrocellulose membrane by semi-wet transfer with 1× Power Blotter 1-Step Transfer Buffer (Thermo Fisher Scientific) following the manufacturer’s instructions. After transfer, the membranes were stained with 0.1% Ponceau S (dissolved in 5% acetic acid) and visualized with a ChemiDoc MP imager (Bio-Rad, Hercules, CA, USA) for subsequent normalization to total protein. The membranes were blocked in a 5% dry milk solution for 1 h. After three washes with PBST, they were incubated (overnight at 4 °C) in PBST solution containing 0.05% NaN_3_ and one of the following primary antibodies: rabbit anti-GFAP (1:20,000, ab7260, Abcam), rabbit anti-EAAT2 (1:3 000, ab205248, Abcam), rabbit anti-GluN2a (1:1000, ab169873, Abcam), rabbit anti-GluN2b (1:1000, ab65783, Abcam), rabbit anti-GluA1 (1:10,000, ab109450, Abcam), or mouse anti-GluA2 (1:7500, MAB397, Sigma-Aldrich). Then, membranes were washed with PBST six times for 5 min each and incubated (room temperature for 1 h) in the secondary antibody solution (1:60,000 goat anti-rabbit IgG-HRP, Pierce, 31460, Thermo Fisher Scientific or 1:80,000 goat anti-mouse IgG-HRP, Pierce, 31430, Thermo Fisher Scientific) in PBST containing 5% dry milk. After three washes with PBST, proteins were detected with SuperSignal™ West Pico PLUS Chemiluminescent Substrate (Thermo Fisher Scientific) and visualized with a ChemiDoc MP imager (Bio-Rad). The same membrane was used to detect the GluA1 and GluA2 signals. After visualization of GluA2, the membrane was incubated in 30% hydrogen peroxide solution for 10 min at 65 °C [95]. The absence of a signal was detected by a similar visualization method. For GluA1 visualization, all steps were repeated, starting with milk incubation for 1 h. Images were analyzed using Image Lab 6.0.1 software (Bio-Rad). Protein expression was normalized to the total protein loading of Ponceau-stained membranes [96] following a total protein normalization method (Bio-Rad). The ratio of the optical densities of the specific protein band to total protein was normalized to the calibrator sample.

### 4.10. Statistical Analysis

Statistical analysis was performed with SPSS Statistics 23 (IBM, Armonk, New York, NY, USA) and StatSoft Statistica 8 (TIBCO, Palo Alto, CA, USA). For the survival analysis, we used the Kaplan–Meier procedure with Breslow’s criterion to test distribution equality. Iglewicz and Hoaglin’s robust test for multiple outliers was used for the identification and rejection of outliers. The Kolmogorov–Smirnov and Shapiro–Wilk tests were used to examine the normality of distribution. Student’s *t*-test, one-way or repeated measures ANOVA with Tukey’s post hoc test were used for normally distributed data. Welch’s ANOVA and the Games–Howell post hoc test were used if the assumption of homogeneity of variances was violated. For all tests, group differences were considered statistically significant at the *p* < 0.05 level. All histograms represent means ± SDs unless otherwise specified.

## Data Availability

The data presented in this study are available upon request from the corresponding author.

## Appendix A

**Table A1:** Primers and probes used in RT-qPCR.

Gene Symbol RefSeq Accession Number	Nucleotide Sequences (Forward, Reverse, TaqMan Probe)	Final Primers and Probe Concentration (nM)	Reference
*Gfap* NM_017009.2	TGGCCACCAGTAACATGCAA CAGTTGGCGGCGATAGTCAT HEX-CGGTCCAAGTTTGCAGACCTCACAG-BHQ2	200 200	[97] (primers) [34] (probe)
*Slc1a2* NM_001035233.1	CCAGTGCTGGAACTTTGCCT TAAAGGGCTGTACCATCCAT FAM-AGCGTGTGACCAGATTCGTCCTCCCA-BHQ1	200 150	[98] (primers) [34] (probe)
*Grin1* NM_017010	GTTCTTCCGCTCAGGCTTTG AGGGAAACGTTCTGCTTCCA FAM-CGGCATGCGCAAGGACAGCC-BHQ1	200 100	[99]
*Grin2a* NM_012573	GCTACACACCCTGCACCAATT CACCTGGTAACCTTCCTCAGTGA HEX-TGGTCAATGTGACTTGGGATGGCAA-BHQ2	200 100	[100]
*Grin2b* NM_012574	CCCAACATGCTCTCTCCCTTAA CAGCTAGTCGGCTCTCTTGGTT HEX-GACGCCAAACCTCTAGGCGGACAG-BHQ2	200 100	[100]
*Gria1* NM_031608	TCAGAACGCCTCAACGCC TGTAGTGGTACCCGATGCCA ROX-TCCTGGGCCAGATCGTGAAGCTAGAAAA-BHQ2	200 100	[101]
*Gria2* NM_017261	CAGTGCATTTCGGGTAGGGA TGCGAAACTGTTGGCTACCT FAM-TCGGAGTTCAGACTGACACCCCA-BHQ1	200 100	[101]
*Actb* NM_031144	TGTCACCAACTGGGACGATA GGGGTGTTGAAGGTCTCAAA FAM-CGTGTGGCCCCTGAGGAGCAC-BHQ1	200 200	[102] (primers) [86] (probe)
*Gapdh* NM_017008	TGCACCACCAACTGCTTAG GGATGCAGGGATGATGTTC R6G-ATCACGCCACAGCTTTCCAGAGGG-BHQ2	200 100	[103]
*B2m* NM_012512	TGCCATTCAGAAAACTCCCC GAGGAAGTTGGGCTTCCCATT ROX-ATTCAAGTGTACTCTCGCCATCCACCG-BHQ1	200 100	[104]
*Rpl13a* NM_173340	GGATCCCTCCACCCTATGACA CTGGTACTTCCACCCGACCTC FAM-CTGCCCTCAAGGTTGTGCGGCT-BHQ1	200 100	[105] (primers) [86] (probe)
*Sdha* NM_130428	AGACGTTTGACAGGGGAATG TCATCAATCCGCACCTTGTA R6G-ACCTGGTGGAGACGCTGGAGCT-BHQ2	200 100	[106] (primers) [86] (probe)
*Ppia* NM_017101	AGGATTCATGTGCCAGGGTG CTCAGTCTTGGCAGTGCAGA ROX-CACGCCATAATGGCACTGGTGGCA-BHQ1	200 100	[61]
*Hprt1* NM_012583	TCCTCAGACCGCTTTTCCCGC TCATCATCACTAATCACGACGCTGG FAM-CCGACCGGTTCTGTCATGTCGACCCT-BHQ1	200 100	[107] (primers) [86] (probe)
*Pgk1* NM_053291	ATGCAAAGACTGGCCAAGCTAC AGCCACAGCCTCAGCATATTTC R6G-TGCTGGCTGGATGGGCTTGGA-BHQ2	200 100	[108] (primers) [86] (probe)
*Ywhaz* NM_013011	GATGAAGCCATTGCTGAACTTG GTCTCCTTGGGTATCCGATGTC ROX-TGAAGAGTCGTACAAAGACAGCACGC-BHQ1	200 100	[108] (primers) [86] (probe)
